# Effect of Hangeshashin-To (Japanese Herbal Medicine Tj-14) on Tolerability of Irinotecan: Propensity Score and Instrumental Variable Analyses

**DOI:** 10.3390/jcm7090246

**Published:** 2018-08-28

**Authors:** Hirokazu Urushiyama, Taisuke Jo, Hideo Yasunaga, Nobuaki Michihata, Hayato Yamana, Hiroki Matsui, Wakae Hasegawa, Yoshihisa Hiraishi, Akihisa Mitani, Kiyohide Fushimi, Takahide Nagase, Yasuhiro Yamauchi

**Affiliations:** 1 Department of Respiratory Medicine, Graduate School of Medicine, The University of Tokyo, 7-3-1 Hongo, Bunkyo-ku, Tokyo 113-8655, Japan; hiro-urushi@umin.ac.jp (H.U.); wakaetnk0703@yahoo.co.jp (W.H.); blueeyedsoul1980@gmail.com (Y.H.); mitania-tky@umin.ac.jp (A.M.); takahide-tky@umin.ac.jp (T.N.); yas-kkr@umin.ac.jp (Y.Y.); 2 Department of Health Services Research, Graduate School of Medicine, The University of Tokyo, 7-3-1 Hongo, Bunkyo-ku, Tokyo 113-8655, Japan; michihata-tky@umin.org (N.M.); yamana-tky@umin.ac.jp (H.Y.); 3 Department of Clinical Epidemiology and Health Economics, School of Public Health, The University of Tokyo, 7-3-1 Hongo, Bunkyo-ku, Tokyo 113-8655, Japan; yasunagah-tky@umin.ac.jp (H.Y.); ptmatsui-tky@umin.ac.jp (H.M.); 4 Department of Health Policy and Informatics, Tokyo Medical and Dental University Graduate School of Medicine, 1-5-45 Yushima, Bunkyo-ku, Tokyo 113-8510, Japan; kfushimi.hci@tmd.ac.jp

**Keywords:** Hangeshashin-to (TJ-14), irinotecan, cancer chemotherapy, drug intolerance, supportive therapy, cohort study, clinical epidemiology, propensity score, inverse probability treatment weighting, instrumental variable

## Abstract

Irinotecan hydrochloride (CPT-11) is used to treat a wide spectrum of malignant tumors. Hangeshashin-to (Japanese herbal medicine TJ-14) is reportedly effective in preventing and controlling diarrhea associated with CPT-11. However, the effect of TJ-14 on tolerability of chemotherapy with CPT-11 has not been fully investigated. We used the Japanese Diagnosis Procedure Combination inpatient database to retrospectively identify patients who had received CPT-11 on their first admission with and without TJ-14. Patients who did receive TJ-14 (*N* = 7092) received CPT-11 more often and in larger doses than those who did not receive TJ-14 (*N* = 82,019). The incidence rate ratio of CPT-11 administration was 1.34 for frequency (95% confidence interval [CI], 1.31–1.38; *p* < 0.001), and 1.16 for total dose (95% CI, 1.14–1.19; *p* < 0.001) according to stabilized inverse probability treatment weighting using propensity scores. Instrumental variable analysis showed similar trends. In-hospital mortality was significantly lower in patients who received TJ-14 than in those who did not. Odds ratios of in-hospital death in patients receiving TJ-14 was 0.81 (95% CI, 0.71–0.93; *p* = 0.002) according to stabilized inverse probability treatment weighting using propensity scores and 0.42 (95% CI, 0.22–0.81; *p* = 0.009) according to instrumental variable analysis. Our findings indicate that TJ-14 improve the tolerability of CPT-11.

## 1. Introduction

Irinotecan hydrochloride (CPT-11), a semisynthetic derivative of camptothecin, is an anticancer drug that inhibits nucleic acid synthesis by topoisomerase I inhibition [1]. CPT-11 possesses a wide antitumor spectrum. However, diarrhea, a characteristic adverse effect of CPT-11, is often severe and a major reason for discontinuing it. The diarrhea in patients receiving CPT-11 is caused by enterocolitis associated with retained SN-38, which is an active metabolite of CPT-11, in the intestine [2]. Most SN-38 is inactivated by formation of SN-38-glucuronide conjugates and excreted in the bile. However, SN-38-glucuronide conjugate is deconjugated with β-glucuronidase from intestinal microflora [3] to SN-38, which cause diarrhea [2].

Kampo medicine (Japanese herbal medicine) Hangeshashin-to (TJ-14), which contains baicalein, is prepared from seven medicinal herbs including *Pinelliae tuber*, *Scutellariae radix*, *Glycyrrhizae radix*, *Zizyphi fructus, Ginseng radix*, *Coptidis rhizome*, and *Zingiberis siccatum rhizoma*. TJ-14 is generally prescribed to treat gastrointestinal diseases such as diarrhea and gastroenteritis in Japan. Baicalein, an inhibitor of β-glucuronidase, inhibits deconjugation of SN-38-glucuronide conjugate and ameliorates diarrhea induced by CPT-11 in rats [4]. TJ-14 was reportedly effective in preventing and controlling diarrhea associated with CPT-11 in 41 patients with non-small cell lung cancer [5]. However, this early study was small and did not include patients with malignancies other than non-small cell lung cancer. Whether TJ-14 improves tolerability of CPT-11 has thus not yet been clearly determined.

In this study, we aimed to examine the influence of TJ-14 on tolerability of CPT-11 by comparing in-hospital mortality and dosage in early courses of chemotherapy with CPT-11 between patients who did and did not receive TJ-14, data being extracted from a national inpatient database in Japan.

## 2. Materials and Methods

### 2.1. Data Source

In this retrospective cohort study, data were extracted from the Diagnosis Procedure Combination database [6], a national inpatient database that includes the following: patient age, sex, body height and weight, primary diagnosis, TNM classification, Charlson comorbidity index, activities of daily living scores, medications during hospitalization, discharge status, and prefecture codes. This study was approved by the Institutional Review Board of The University of Tokyo, which waived the requirement for informed patient consent because of the anonymous nature of the data, (ethic code: 3501).

In this study, the first episode of hospitalization for chemotherapy with CPT-11 treatment during the study period was evaluated, the intention being to evaluate the earliest course, ideally the first course, of chemotherapy with CPT-11.

### 2.2. Patient Selection

Data on patients who had been treated with CPT-11 with a diagnosis of neoplasm defined by the International Statistical Classification of Diseases and Related Health Problems-10th revision (ICD-10) codes between July 2010 and March 2016 were collected. Patients who had received TJ-14 before or on the day of initial CPT-11 administration during hospitalization were defined as in the TJ-14 group and the remaining patients were defined as in the control group.

### 2.3. Variables

The following variables were studied: age, sex, body mass index (kg/m^2^), activities of daily living scores, and items in the TNM staging system. The following cancer types, for which CPT-11 has been approved by the Ministry of Heath, Labour and Welfare were identified: colon cancer (ICD-10 codes, C18, C19, C20), lung cancer (C34), gastric cancer (C16), ovarian cancer (C56), pancreatic cancer (C25), non-Hodgkin lymphoma (C82, C83, C85, B212), breast cancer (C50), skin cancer (C44, C519, C609, C632), and other cancers. The following comorbidities were also identified using ICD-10 codes: chronic obstructive pulmonary disease (J41, J42, J43, J44), interstitial pneumonia (B221, J701, J704, J841, J848, J849, J990, J991, M321, M330, M331, M332, M351), congestive heart failure (E059, I46, I50, I099, I110), ischemic heart disease (I20-25), tachycardia (I47-49, R000, T818), pulmonary thromboembolism (I26), chronic liver disease (B89, B181, B182, B659, B661, K702, K703, K72, K74, K761, K762, K763, K766, K767), chronic renal failure (E102, E112, E142, I120, N17-19), autoimmune diseases (M05, M06, M08, M30-35), mycosis (A420, A43, B37, B380-382, B390-392, B400-402, B410, B420, B440, B441, B449, B460, J172), and bacterial pulmonary infection (A481, J100, J110, J12-16, J170, J178, J18, J85, J86). Procedures and treatments during hospitalization, including surgery with general anesthesia, intensive care unit hospitalization, mechanical ventilation, and hemodialysis were also examined, as was use of drugs that can be used to treat diarrhea, including loperamide hydrochloride, berberine chloride hydrate, and lactomin. Use of drugs that can be used to treat or prevent myelosuppression, such as filgrastim, lenograstim, nartograstim, and pefilgrastim was also examined.

### 2.4. Primary Outcomes

The outcomes of this study were (i) in-hospital death, and (ii) frequency and total amount of CPT-11 administered during hospitalization.

### 2.5. Statistical Analyses

Continuous variables are presented as mean and standard deviation (SD) or median with interquartile range (IQR). Mann-Whitney U tests were performed to compare frequencies and total amount of CPT-11 between patients who did and did not receive TJ-14. In-hospital deaths were compared between patients who did and did not receive TJ-14 using Pearson’s χ^2^ test. (i) Stabilized inverse probability of weighting (IPTW) analyses using propensity scores and (ii) instrumental variable (IV) analyses were performed to compare the outcomes between patients who did and did not receive TJ-14 to account for differences in each individual patient’s background characteristics.

Stabilized IPTW employs propensity scores and adjusts for measured potential confounding [7] while preserving sample size. To control for imbalance in covariates, patient’s specific stabilized weights were generated using propensity scores, which predict probability of initiating TJ-14. Balance of covariates was assessed by using standardized mean difference; <0.1 indicates acceptable balancing of covariates between the two groups. Stabilized IPTW analyses preserves sample size and appropriately estimates average treatment effects over marginal distribution of measured covariates in a study cohort. To estimate the propensity score, a logistic regression model for receiving TJ-14 as a function of relevant patient characteristics was constructed. These characteristics included age, sex, body mass index, activities of daily life scale (Barthel index), cancer types, T, N and M factors, medicines for treating diarrhea and febrile neutropenia, surgery with general anesthesia, intensive care unit hospitalization, mechanical ventilation, hemodialysis, comorbidities, season of admission, and geographical area of residence.

IV analyses were conducted to confirm the results of stabilized IPTW analyses because hidden biases caused by unmeasured confounders are not removed with the stabilized IPTW method. The IV method is another means of creating a pseudorandomized situation and technically enables controlling for unmeasured confounders. The followings are the key assumptions of the IV: (i) the IV is highly correlated with the treatment assigned; (ii) it is not correlated with any measured or unmeasured patient characteristics; and (iii) it does not affect patient outcomes except through the studied treatment [8]. In general, individual physicians and hospitals have preferences for therapy for managing adverse drug reaction caused by anticancer agents. When use of herbal medicine such as TJ-14 is strongly consistent within a hospital, decisions about supportive therapy for anticancer agents is presumably made independently of individual patient’s characteristics. Thus, for such hospitals, using TJ-14 depends more on the hospital at which a patient is treated than on that patient’s specific risk factors. Under these circumstances, the hospital’s preference for herbal medicine as supportive therapy for anticancer agents was considered to act as an IV, even in the presence of unmeasured confounders. In this study, “the percentage of TJ-14 use in each hospital”, which was calculated by dividing the number of patients receiving TJ-14 by the number receiving CPT-11, was used as an IV. The F-test was used to identify weak instrument; F-statistic <10 being considered to denote a weak IV [9]. To ensure for robustness, this IV was used in a two stages residual inclusion method to compute incidence rate ratios of frequency and total amount of CPT-11 administered and the odds ratio of in-hospital death between the two groups in a generalized liner model. Logistic regression was conducted for in-hospital death and Poisson regression for frequency and total amount of CPT-11 administered in both propensity-score stabilized IPTW and IV analyses.

All statistical analyses were performed using SPSS version 23.0 (IBM SPSS, Armonk, NY, USA) and Stata version 15.1 (StataCorp, College Station, TX, USA). A two tailed significance level of 0.05 was used in all statistical analysis.

## 3. Results

### 3.1. Patient Characteristics

We identified 90,437 patients who had received chemotherapy with CPT-11 and 7239 who had received TJ-14 before or on the same day they received CPT-11. The baseline characteristics of all patients (*N* = 90,437) and patients after stabilization by IPTW estimation (*N* = 89,111) are shown in Table 1. Among all patients, larger proportions of patients with lung and ovarian cancer received TJ-14, whereas smaller proportions of patients with colon and gastric cancer received TJ-14. We have provided detailed information on cancer types of all patients in Supplementary Table S1. We corrected these imbalances in baseline patient characteristics by stabilized IPTW estimation using propensity scores.

Comorbidities on first admission for chemotherapy with CPT-11 are presented in Table 2. There were more patients with chronic obstructive pulmonary disease in the TJ-14 group; we also corrected this imbalance by stabilized IPTW estimation.

### 3.2. Tolerability of CPT-11

To examine the tolerability of CPT-11, we analyzed the dosage of CPT-11 administered in TJ-14 and control groups. In all patients, mean frequency and amount of CPT-11 administered on first admission were significantly greater in the TJ-14 than the control group. Mean frequencies were 1.55 (SD 1.31) in the control group and 2.22 (SD 2.06) in the TJ-14 group (*p* < 0.001). Mean total doses (milligrams) were 222 (SD 143) in the control group and 262 (SD 222) in the TJ-14 group (*p* < 0.001). The generalized linear model in patients after stabilized IPTW estimation showed that the incidence rate ratio of CPT-11 administration was 1.34 (95% confidence interval (CI), 1.31–1.38; *p* < 0.001) for frequency and 1.16 (95% CI, 1.14–1.19; *p* < 0.001) for total dose. To remove hidden biases caused by unmeasured confounders, we confirmed the results of tolerability of CPT-11 by IV analysis. Our IV analysis had an F-statistic of 4352 with *p* < 0.001. The incidence rate ratio of CPT-11 administration was 1.11 (95% CI, 1.05–1.17; *p* < 0.001) for frequency, and 1.12 (95% CI, 1.11–1.12; *p* < 0.001) for total dose. These results are shown in Table 3.

### 3.3. Mortality during First Admission for Administration of CPT-11

Among all patients, 3972 (4.8%) in the control and 336 (4.6%) in the TJ-14 group died during the first observed hospitalization for chemotherapy with CPT-11. Crude mortality did not differ significantly between the two groups (*p* = 0.26). However, after stabilized IPTW estimation, mortality was significantly lower in the TJ-14 (4.0%) than the control group (4.8%) (*p* = 0.001). In stabilized IPTW analysis, the odds ratio of in-hospital mortality in the TJ-14 group was 0.81 (95% CI, 0.71–0.93; *p* = 0.002) compared with the control group. In IV analysis, the odds ratio of in-hospital mortality in the TJ-14 group was 0.42 (95% CI, 0.22–0.80; *p* = 0.009) compared with the control group. Table 4 shows in-hospital mortality on first observed hospitalization for chemotherapy with CPT-11 with or without TJ-14.

## 4. Discussion

The current study showed that administration of TJ-14 is significantly associated with decreased mortality and increased dosage of first CPT-11 during hospitalization. To the best of our knowledge, this is the first published evidence that TJ-14 improves the tolerability of CPT-11 in a nationwide clinical setting.

In earlier studies, baicalein was found to inhibit β-glucuronidase activity with SN-38-glucuronide as substrate in vitro [10]. TJ-14 and baicalein inhibited deconjugation of SN-38-glucuronide and ameliorated diarrhea symptoms induced by CPT-11 in an animal model [4]. The results of these earlier studies suggest that TJ-14 and baicalein are effective in improving tolerability of CPT-11. TJ-14 is recommended for patients receiving chemotherapy with CPT-11 in a Japanese stewardship guide. However, in clinical settings, TJ-14 has been reported to be effective against CPT-11 toxicity in only 41 patients with non-small cell lung cancer [5]. Consequently, thus far, TJ-14 has only been used in Japan in patients receiving chemotherapy with CPT-11. The current study is the first to provide evidence that TJ-14 significantly improves the tolerability of CPT-11 regardless of cancer type on the basis of retrospectively examining data for a large nationwide clinical cohort.

We conducted a quasi-experimental study using two different pseudorandomization techniques (IPTW analysis using propensity scores and IV analysis) to examine the efficacy of TJ-14 against CPT-11 toxicity. Performing IV analysis allowed us to control for hidden biases caused by unmeasured confounders that are not removed by IPTW or a generalized linear model. IV analysis also showed that TJ-14 had a beneficial effect on mortality with a lower point estimate of risk compared with propensity score analysis. IV analysis only evaluates the effectiveness of the treatment in a patient group changing treatment according to the instrumental variable (i.e., local average treatment effect) [11]. This effect may thus be strong because it was small that the number of patients who might have received TJ-14 if they had been in a hospital that tended to use TJ-14 with CPT-11, according to the IV. Taken together, we consider that the demonstrated efficacy of TJ-14 against CPT-11 toxicity in the current study provides robust evidence for using it as supportive therapy in patients receiving CPT-11.

In this study, we were not able to take into account the severity of adverse effects of CPT-11; e.g., diarrhea and febrile neutropenia. We, therefore, did not evaluate the incidence of diarrhea or febrile neutropenia as endpoints of this study. Instead, we evaluated prescription of antidiarrheal agents (loperamide and lactobacillus preparations) and the use of granulocyte colony-stimulating factor as variables in stabilized IPTW and IV analysis. We believe that our stabilized IPTW and IV analysis did thereby adjust for these adverse effects, thus accurately evaluating the efficacy of TJ-14 against CPT-11 toxicity.

This study has some limitations. First, we could not determine therapies administered prior to chemotherapy with CPT-11 or causes of in-hospital deaths. However, we believe that the effects of prior therapies or causes of death would have been adjusted for by the IV analysis. Our study thus may have accurately evaluated the efficacy of TJ-14. Second, we were also unable to take into consideration genetic polymorphisms on UDP-glucuronosyl transferase 1A1 (UGT1A1), which predominantly inactivates SN-38 to form a glucuronide metabolite. About 10% of patients with reduced UGT1A1 activity are at increased risk for diarrhea and neutropenia [12,13]. However, it is unlikely that UGT1A1 activity biased the selection of treatment with TJ-14 in patients receiving CPT-11. Third, we evaluated only the initial hospitalization episode for each patient treated with CPT-11 with the intent of capturing the first course of chemotherapy with CPT-11 because the dosage or intervals between administrations of CPT-11 may have been reduced or prolonged because of adverse drug reactions in subsequent courses. However, we may not have succeeded in capturing the first course of chemotherapy with CPT-11 in some cases. For example, patients who had switched hospitals for subsequent courses of a CPT-11 regimen, started a CPT-11 regimen as an outpatient but subsequently been hospitalized (e.g., because of an adverse drug reaction), or had been in hospitals that started to provide DPC data after initiation of CPT-11 may have erroneously been treated as though the studied hospitalization for administration of CPT-11 was the first. However, it is likely that most assessed episodes were for the first course of a CPT-11 regimen. Moreover, the reason for not evaluating outpatients was that environmental factors that may affect outcomes can be minimized in hospitalized patients.

## 5. Conclusions

The present study of data extracted from a nationwide database and examined using two pseudorandomization techniques, stabilized IPTW and IV, provides evidence that TJ-14 improves the tolerability of CPT-11 in hospitalized patients. We therefore recommend TJ-14 as a useful supportive therapy for patients receiving CPT-11.

## Figures and Tables

**Table 1:** Baseline characteristics of patients on first admission for CPT-11 chemotherapy with or without TJ-14, before and after stabilized inverse probability treatment weighting using propensity scores.

Characteristic (Categorical)	All Patients					Patients after IPTW Estimation				
	Control		TJ-14			Control		TJ-14		
	(*N* = 83,198)		(*N* = 7239)			(*N* = 82,019)		(*N* = 7092)		
	*N*	%	*N*	%	s.d.	*N*	%	*N*	%	s.d.
Sex (male)	49,417	59.4	4347	60.1	0.012	48,732	59.4	4195	59.2	0.006
Cancer type ^a^										
Colon cancer	36,595	44.0	1937	26.8	−0.37	34,958	42.6	3004	42.4	−0.006
Lung cancer	14,271	17.2	2438	33.7	0.39	15,165	18.5	1351	19.1	0.013
Gastric cancer	11,078	13.3	571	7.9	−0.18	10,560	12.9	914	12.9	0.000
Ovarian cancer	4438	5.3	589	8.1	0.11	4586	5.6	408	5.8	0.006
Pancreatic cancer	4421	5.3	317	4.4	−0.044	4310	5.3	359	5.1	−0.009
Cervical cancer	4039	4.9	452	6.2	0.058	4074	5.0	356	5.0	0.002
Non-Hodgkin lymphoma	1042	1.3	170	2.4	0.083	1093	1.3	100	1.4	0.006
Breast cancer	311	0.4	28	0.4	0.005	300	0.4	23	0.3	−0.007
Skin cancer	88	0.1	26	0.4	0.051	105	0.1	9	0.1	−0.000
Other cancer	7098	8.5	736	10.2	0.057	7057	8.6	581	8.2	−0.014
Activities of daily life (Barthel index)										
Independent (100)	72,572	87.2	6293	86.9	−0.007	71,717	87.4	6184	87.2	−0.008
Dependent (≤95)	7638	9.2	675	9.3	0.003	7463	9.1	676	9.5	0.015
Missing	2988	3.6	271	3.7	0.009	2839	3.5	232	3.3	−0.010
Characteristic (numerical)	Mean	S.D.	Mean	S.D.	s.d.	Mean		Mean		s.d.
Age (years)	64.5	10.9	64.0	11.3	−0.004	64.4		64.6		0.015
Body mass index (kg/m2)	21.9	3.7	22.1	3.8	0.063	21.9		21.9		0.001

^a^ overlapping of cancer types, detailed information in Supplementary Materials; IPTW, inverse probability treatment weighting; s.d., standardized difference; S.D., standard deviation.

**Table 2:** Comorbidities on first admission for CPT-11 chemotherapy with or without TJ-14, before and after stabilized inverse probability treatment weighting using propensity scores.

	All Patients					Patients after IPTW Estimation				
	Control		TJ-14			Control		TJ-14		
	(*N* = 83,198)		(*N* = 7239)			(*N* = 82,019)		(*N* = 7092)		
	*N*	%	*N*	%	s.d.	*N*	%	*N*	%	s.d.
Chronic obstructive pulmonary disease	2611	3.1	387	5.4	0.11	2725	3.3	236	3.3	0.001
Interstitial pneumonia	477	0.6	66	0.9	0.041	492	0.6	43	0.6	0.000
Congestive heart failure	1996	2.4	188	2.6	0.013	1347	2.4	117	2.4	−0.003
Ischemic heart disease	2571	3.1	278	3.8	0.040	2587	3.2	238	3.4	0.011
Tachycardia	1937	2.3	209	2.9	0.036	1947	2.4	172	2.4	0.003
Chronic liver disease	1554	1.9	134	1.9	−0.001	1525	1.9	142	2.0	0.010
Chronic renal failure	1224	1.5	123	1.7	0.020	1225	1.5	98	1.4	−0.009
Autoimmune disease	585	0.7	61	0.8	0.017	586	0.7	52	0.7	0.002

IPTW, inverse probability treatment weighting; s.d., standardized difference.

**Table 3:** Incidence rate ratios of CPT-11 administration with TJ-14 compared with without TJ-14 by generalized linear models and instrumental variable analyses.

CPT-11 Administration	Generalized Linear Model			Instrumental Variable Analysis		
	IRR	95% CI	*p*	IRR	95% CI	*p*
frequency	1.34	1.31–1.38	<0.001	1.11	1.05–1.17	<0.001
amount	1.16	1.14–1.19	<0.001	1.12	1.11–1.12	<0.001

IRR, incidence rate ratio; CI, confidence interval.

**Table 4:** In-hospital death on first admission for CPT-11 administration with or without TJ-14.

	All Patients					Patients after IPTW Estimation					
	Control		TJ-14			Control		TJ-14		
	(*N* = 83,198)		(*N* = 7239)			(*N* = 82,019)		(*N* = 7092)		
	*N*	%	*N*	%	*p*	*N*	%	*N*	%	*p*
In-hospital death	3972	4.8	336	4.6	0.26	3936	4.8	280	4.0	0.001
	Generalized linear model					Instrumental variable analysis					
	Odds ratio		95% CI		*p*	Odds ratio		95% CI		*p*
In-hospital mortality of TJ-14 compared with the control	0.81		0.71–0.93		0.002	0.42		0.22–0.80		0.009

IPTW, inverse probability treatment weighting; CI, confidence interval.

## Supplementary Materials

The following are available online at http://www.mdpi.com/2077-0383/7/9/246/s1, Table S1: Detailed information of cancer types of all patients.
