# Supplementary material for: Effect of Hangeshashin-To (Japanese Herbal Medicine Tj-14) on Tolerability of Irinotecan: Propensity Score and Instrumental Variable Analyses
Source: J Clin Med. 2018 Aug 28;7(9):246. doi: 10.3390/jcm7090246 (PMC6162861; doi:10.3390/jcm7090246)
Supplement: Supplementary file 1 [file jcm-07-00246-s001.zip › jcm-347755-SI.doc]

**Table S1: Detailed information of cancer types of all patients**

|  | All patients | | | |
| --- | --- | --- | --- | --- |
|  | Control | | TJ-14 | |
|  | (*N*=83198) | | (*N*=7239) | |
| Cancer type | *N* | % | *N* | % |
| Colon cancer | 36498 | 43.9 | 1934 | 26.7 |
| Lung cancer | 14186 | 17.1 | 2418 | 33.4 |
| Gastric cancer | 11010 | 13.2 | 565 | 7.8 |
| Ovarian cancer | 4423 | 5.3 | 587 | 8.1 |
| Pancreas cancer | 4371 | 5.3 | 308 | 4.3 |
| Cervical cancer | 4025 | 4.8 | 449 | 6.2 |
| Non-Hodgkin lymphoma | 1025 | 1.2 | 168 | 2.3 |
| Breast cancer | 298 | 0.4 | 24 | 0.3 |
| Skin cancer | 82 | 0.1 | 26 | 0.4 |
| Other cancer | 7098 | 8.5 | 736 | 10.2 |
| Colon and gastric cancer | 35 | 0.0 | 0 | 0.0 |
| Colon and lung cancer | 29 | 0.0 | 2 | 0.0 |
| Lung and pancreas cancer | 20 | 0.0 | 8 | 0.1 |
| Lung and gastric cancer | 15 | 0.0 | 4 | 0.1 |
| Colon and pancreas cancer | 15 | 0.0 | 0 | 0.0 |
| Gastric and pancreas cancer | 13 | 0.0 | 1 | 0.0 |
| Lung and Non-Hodgkin lymphoma | 10 | 0.0 | 1 | 0.0 |
| Colon and ovarian cancer | 7 | 0.0 | 0 | 0.0 |
| Lung and breast cancer | 7 | 0.0 | 2 | 0.0 |
| Ovarian and cervical cancer | 5 | 0.0 | 1 | 0.0 |
| Colom and breast cancer | 4 | 0.0 | 1 | 0.0 |
| Colon and cervical cancer | 3 | 0.0 | 0 | 0.0 |
| Colon and Non-Hodgkin lymphoma | 3 | 0.0 | 0 | 0.0 |
| Cervical and skin cancer | 3 | 0.0 | 0 | 0.0 |
| Lung and cervical cancer | 2 | 0.0 | 2 | 0.0 |
| Gastric and Non-Hodgkin lymphoma | 2 | 0.0 | 1 | 0.0 |
| Ovarian and breast cancer | 1 | 0.0 | 0 | 0.0 |
| Gastric and ovarian cancer | 1 | 0.0 | 0 | 0.0 |
| Pancreas and ovarian cancer | 1 | 0.0 | 0 | 0.0 |
| Breast and cervical cancer | 1 | 0.0 | 0 | 0.0 |
| Lung and skin cancer | 1 | 0.0 | 0 | 0.0 |
| Gastric and skin cancer | 1 | 0.0 | 0 | 0.0 |
| Colon and skin cancer | 1 | 0.0 | 0 | 0.0 |
| Lung, gastric, and Non-Hodgkin lymphoma | 1 | 0.0 | 0 | 0.0 |
| Pancreas and Non-Hodgkin lymphoma | 1 | 0.0 | 0 | 0.0 |
| Lung, ovarian, and breast cancer | 0 | 0.0 | 1 | 0.0 |
